# The influence of a caveolin-1 mutant on the function of P-glycoprotein

**DOI:** 10.1038/srep20486

**Published:** 2016-02-04

**Authors:** Chih-Yuan Lee, Ting-Yu Lai, Meng-Kun Tsai, Pu Ou-Yang, Ching-Yi Tsai, Shu-Wei Wu, Li-Chung Hsu, Jin-Shing Chen

**Affiliations:** 1 Department of Surgery, No. 7 Chung San South Road, Taipei 10002, Taiwan; 2 Department of Medical Research, National Taiwan University Hospital, No. 7 Chung San South Road, Taipei 10002, Taiwan; 3 Institute of Molecular Medicine, College of Medicine, National Taiwan University, No. 1 Jen-Ai Road, Sec. 1, Taipei 10002, Taiwan

## Abstract

The genetic heterogeneity in cancer cells has an increased chance in the acquisition of new mutant such as drug-resistant phenotype in cancer cells. The phenotype of drug resistance in cancer cells could be evaluated by the number or function of drug transporters on cell membranes, which would lead to decreased intracellular anti-cancer drugs concentration. Caveolae are flask-shaped invaginations on cell membrane that function in membrane trafficking, endocytosis, and as a compartment where receptors and signaling proteins are concentrated. Caveolin-1 (CAV1) is the principal structural protein of caveolae and closely correlates with multidrug resistance in cancer cells. In a systematic study of the ubiquitin-modified proteome, lysine 176 of CAV1 was identified as a potential post-translational modification site for ubiquitination. In this article, we identified a mutation at lysine 176 to arginine (K176R) on CAV1 would interfere with the biogenesis of caveolae and broke the interaction of CAV1 with P-glycoprotein. Functional assays further revealed that K176R mutant of CAV1 in cancer cells increased the transport activity of P-glycoprotein and decreased the killing ability of anti-cancer drugs in non-small-cell lung cancer cell lines.

The plasma membrane of cells consists not only of a uniform phospholipid bilayer but also of a dynamic assembly of a variety of different lipids and proteins, including small (10–200 nm) sphingolipid- and cholesterol-enriched components termed “lipid rafts”^1^. Caveolae are flask-shaped invaginations on the lipid rafts that function in membrane trafficking, endocytosis, and as a compartment in which receptors and signaling proteins are concentrated to amplify specific signaling cascades. Caveolin-1 (CAV1) is the principal structural protein of caveolae and might function as a scaffolding protein to organize membrane signaling proteins within these structures^2^. CAV1 was initially identified as a 178-amino-acid (24 kDa) protein that forms oligomers at the plasma membrane, which are structural and functional elements of caveolae^3^. CAV1 interacts with itself to form homo-oligomers, and these oligomer/oligomer interactions then produce an interlocking network of CAV1 molecules that contribute to the basic structure of caveolae^4^,^5^.

In a systematic study of the ubiquitin-modified proteome, lysine 176 of CAV1 was identified as a potential post-translational modification site for ubiquitination. However, the function of CAV1 ubiquitination at lysine 176 remains unknown^6^.

Chemotherapy primarily fails due to the emergence of cellular resistance to anti-cancer drugs. After exposure to an anti-cancer drug, cancer cells can become simultaneously insensitive to unrelated drugs. This phenomenon is called multidrug resistance (MDR). Some studies have shown that CAV1 expression closely correlates with the development of MDR in cancer cells. High levels of CAV1 were observed in a number of MDR cancer cell lines, such as adriamycin-resistant MCF-7 breast adenocarcinoma cells and colchicine-resistant HT-29 colon carcinoma cells^7^. However, an increasing number of studies indicate that an elevated level of CAV1 is not the only cause of MDR^8^,^9^. Therefore, we hypothesized that the post-translational modification of CAV1 might contribute to the emergence of MDR in cancer cells.

MDR is a serious problem in chemotherapy for cancers. Several ATP-binding cassette (ABC) efflux transporters that pump anti-cancer drugs out of cancer cells are the main transporters responsible for MDR, such as P-glycoprotein, MDR-associated protein 1 (MRP1/ABCC1) and breast cancer resistance protein (BCRP/ABCG2)^10^.

Although P-glycoprotein is reportedly located in lipid rafts and associated with CAV1^11^,^12^, the influence of the interaction between CAV1 and P-glycoprotein on the development and progression of MDR in cancer cells is largely unknown. In the present study, we found that the post-translational modification site of CAV1 at lysine 176 influenced the formation of CAV1 oligomers and the interaction between CAV1 and P-glycoprotein, which also affected the transport activity of P-glycoprotein in non-small-cell lung cancer cell lines. Our results suggest that the post-translational modification site of CAV1 at lysine 176 influences the drug transport activity of P-glycoprotein and the drug sensitivity of lung cancer cells.

## Results

### Lysine 176 mutation influences the oligomerization of caveolin 1

**Figure 1:**
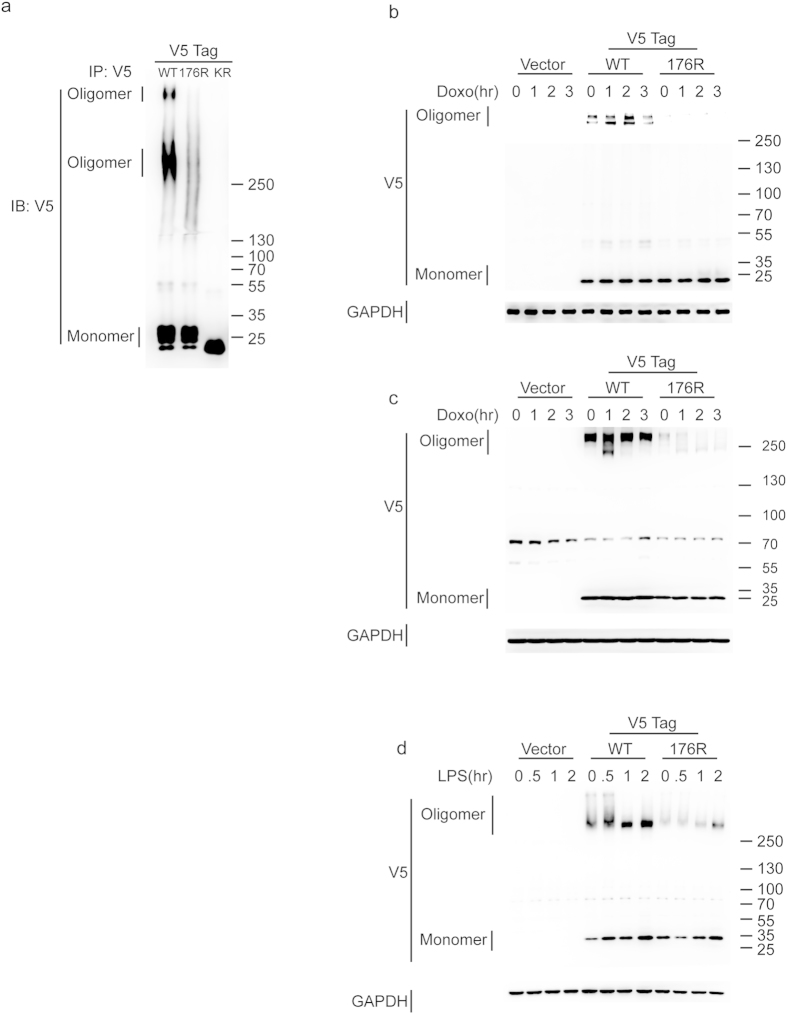
The K176R mutation of CAV1 impaired oligomerization. (**a**) HEK293T cells were transfected with either V5-tagged CAV1 (WT), CAV1 K176R mutant (176R) or CAV1 all K to R mutant (KR), in which all 12 lysines were mutated to arginine and served as a negative control. Oligomerization was determined by immunoprecipitation with anti-V5 antibody. 5–10% gradient SDS PAGE gels were used in the assay. (**b**–**d**) CAV1 or the CAV1 K176R mutant were expressed in the H460 lung cancer, A549 lung carcinoma and RAW264.7 leukemia cell lines by lentiviral delivery, and the cells were separately treated with doxorubicin (0.5 μM) or LPS (100 ng/ml) at the indicated times. Oligomerization of the caveolae was detected using an immunoblot assay and 5–10% SDS PAGE gels were used in the assay.

CAV1, a 178-amino-acid protein that contains 12 lysines in distinct functional domains, is localized in caveolae and acts as an integral membrane protein. It is also a major assembly protein of caveolae. CAV1 contains a central hydrophobic transmembrane domain that is anchored inside the membrane, with both the N and C termini located in the cytosol. According to previous studies, mono-ubiquitin modifies CAV1 at lysines 5 to 65 in the N-terminal domain for vesicle trafficking. However, CAV1 is also ubiquitinated at lysines other than those in the N-terminal region^5^,^13^,^14^. According to the global proteomic analysis performed by Kim *et al.* lysine 176 (K176) could also be the acceptor site for ubiquitin^6^. To identify the function of the ubiquitination of K176 in CAV1, we prepared a V5-tagged lysine 176-to-arginine (K176R) mutant of CAV1 for further analysis. The overexpression of the K176R mutant of CAV1 in HEK293 cells clearly decreased the oligomerization of CAV1 (Fig. 1a). We also transduced the K176R mutant of CAV1 into three other cancer cell lines, including NCI-H460 (non-small-cell lung carcinoma), A549 (non-small-cell lung carcinoma) and RAW264.7 (murine macrophage cell line), using a lentiviral system in order to confirm the impairment of oligomerization of CAV1 in cells transduced with CAV1 K176R mutant. We examined the formation of CAV1 oligomers in NCI-H460 cells and A549 cells after treatment with doxorubicin. The formation of CAV1 oligomers was again significantly decreased both in NCI-H460 and A549 cells that were transduced with the CAV1 K176R mutant (Fig. 1b,c) Lipopolysaccharide (LPS) treatment of macrophages has been reported to induce the expression of CAV1 and enhance the formation of CAV1 oligomers^15^,^16^. Therefore, we transduced either the wild-type or the K176R mutant of CAV1 into RAW264.7 cells and examined the formation of CAV1 oligomers after treatment with LPS. We found that the formation of CAV1 oligomers after LPS treatment significantly decreased in RAW264.7 cells that were transduced with the CAV1 K176R mutant (Fig. 1d). Taken together, these data indicate that the K176R mutant of CAV1 hindered the formation of CAV1 oligomers, which might affect the function of caveolae.

### CAV1 K176R mutation enhances drug resistance via P-glycoprotein

**Figure 2:**
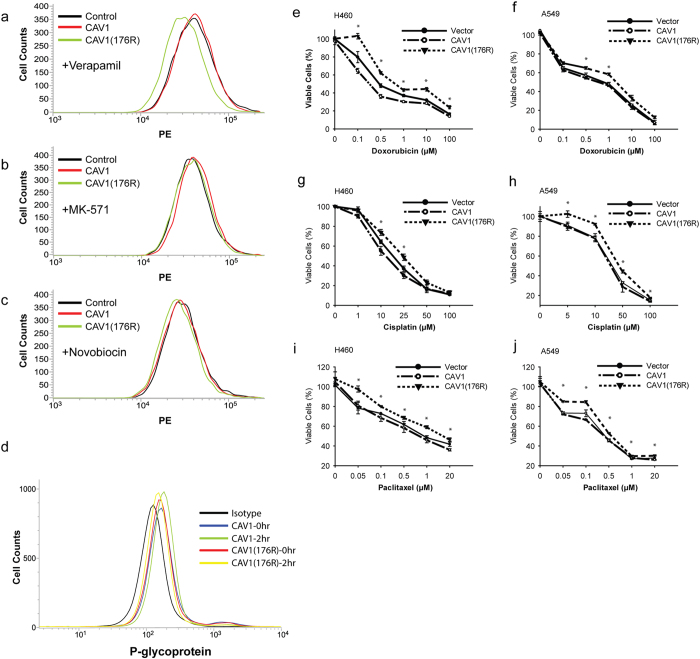
The K176R mutation increased the drug sensitivity of lung cancer cells via P-glycoprotein. (**a**–**c**) Flow cytometry analysis of the intracellular fluorescence intensity of eFluxx-ID Gold in native H460 cells and in cells that were transduced with CAV1 or the CAV1 K176R mutant in the presence of verapamil, MK-571 or novobiocin. (**d**) Surface P-glycoprotein expression in H460 cells that were transduced with CAV1 or the CAV1 K176R mutant were analyzed by flow cytometry. (**e**–**j**) H460 and A549 lung cancer cell lines were cultured in medium containing various concentrations of doxorubicin, cisplatin or paclitaxel for 24 hours. The WST1 assay was performed 24 hours after treatment. The absorbance at 450 nm minus the absorbance at 630 nm was considered to represent the cell density. The values are the average of 4 wells from representative experiments. The experiments were repeated 5 times, and similar results were obtained.

The development of MDR is a serious problem for patients undergoing chemotherapy. Several ABC efflux transporters that pump chemotherapy drugs out of cancer cells participate in MDR, such as P-glycoprotein, MRP1/ABCC1 and BCRP/ABCG2^17^. CAV1 has been shown to contribute to MDR, and abnormal levels of CAV1 can lead to a functional change in multidrug transporters in cancer cells^12^,^18^,^19^. To investigate whether the CAV1 K176R mutant influenced the function of these multidrug transporters, we used an eFluxx-ID Gold MDR assay to quantify the function of the drug transporters by flow cytometry. The eFluxx-ID Gold MDR assay indicated elevated levels of cellular fluorescence due to the trapped fluorescence dye in cells following treatment with multidrug transporter inhibitors. After treatment with MK-571 (a MRP1 inhibitor) or novobiocin (a BCRP inhibitor), the fluorescence intensity did not differ among NCI-H460 cells that were transduced with wild-type CAV1, the CAV1 K176R mutant or the vector control (Fig. 2b,c). However, treatment with verapamil (inhibitor of P-glycoprotein) did not inhibit the function of multidrug transporters in NCI-H460 cells that were transduced with the CAV1 K176R mutant. This finding indicated that the efflux of the eFluxx-ID gold fluorescent dye was increased in cells that were transduced with the CAV1 K176R mutant compared with cells that were transduced with wild-type CAV1 or vector control (Fig. 2a). The inhibitory effect of verapamil, an inhibitor of P-glycoprotein, was not evident in cells that were transduced with the CAV1 K176R mutant. According to the published literature, the overexpression of CAV1 reduces the function of P-glycoprotein^9^,^20^. We examined the surface expression of P-glycoprotein, which did not significantly differ between cells that were transduced with wild-type CAV1 or the K176R mutant, irrespective of doxorubicin treatment (Fig. 2d). These results suggested that the K176R mutant of CAV1 neutralizes the inhibition of P-glycoprotein by CAV1. Doxorubicin, an anti-cancer drug, is one of the substrates that are transported by P-glycoprotein, which decreases the intracellular drug concentrations by transporting the drugs out of the cells and consequently reduces cytotoxicity^21^. To evaluate the cytotoxic effect of doxorubicin in H460 cells that were transduced with either wild-type CAV1, the K176R mutant or the vector control, cells were seeded into microplates and incubated with various concentrations of doxorubicin. The cell viabilities were determined using the WST1 assay. The highest and lowest viabilities were observed for cells that were transduced with the CAV1 K176R mutant and wild-type CAV1 treated with 0.1 to 100 μM doxorubicin, respectively (Fig. 2e). We performed the same experiment in A549 cells, a human lung adenocarcinoma epithelial cell line, and obtained similar results: cells that were transduced with the CAV1 K176R mutant were more viable than wild-type CAV1- or vector-transduced cells (Fig. 2f). Thus, both H460 and A549 cells transduced with the CAV1 K176R mutant are less sensitive to the cytotoxic effects of doxorubicin. Cisplatin, another chemotherapy agent that is used to treat lung cancers, is not a P-glycoprotein substrate. However, cisplatin can induce changes via a P-glycoprotein regulatory pathway, which confers a partial loss of cisplatin cytotoxicity^22^,^23^. To evaluate the effects of the CAV1 K176R mutant on the cytotoxicity of cisplatin, we performed experiments similar to those described above but treated the transduced cells with cisplatin rather than doxorubicin. As shown in Fig. 2g,h similar results were obtained: cells that were transduced with the CAV1 K176R mutant were more viable than cells transduced with either the vector control or wild-type CAV1. Paclitaxel was one of the substrates of the P-glycoprotein. H460 and A549 cells transduced with CAV1 or CAV1 K176R were used to determine the viability of the cells after treatement with paclitaxel. The results were similar to the treatment with doxorubicin or cisplatin and cells transduced with CAV1 K176R mutant possessed higher resistance to anti-cancer drugs (Fig. 2i,j). In summary, both the NCI-H460 and A549 cell lines transduced with the CAV1 K176R mutant were less sensitive to the cytotoxic effects of doxorubicin and cisplatin. The overexpression of CAV1 in cells reportedly inhibits the function of P-glycoprotein^9^,^20^. Therefore, we hypothesized that the CAV1 K176R mutant neutralizes the inhibition of P-glycoprotein by CAV1.

### K176R mutant influences the interaction between CAV1 and P-glycoprotein

**Figure 3:**
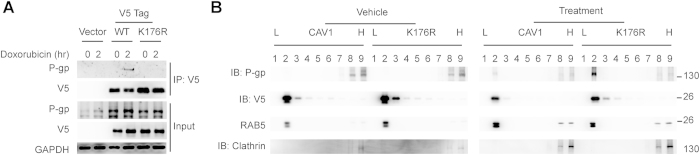
CAV1 K176R mutation impaired the association with P-glycoprotein. (**A**) H460 cells were transduced with either CAV1 or the CAV1 K176R mutant, and the interaction of CAV1 with P-glycoprotein was detected by immunoprecipitation. Cells were treated with doxorubicin (0.5 μM) for the indicated times, followed by immunoblot analysis with the indicated antibodies. (**B**) Total cell extracts from H460 cells that were transduced with either CAV1 or the CAV1 K176R mutant were fractionated on OptiPrep gradients. In the vehicle control group shown in the left panel in Fig. 3B, less P-glycoprotein was present in the lipid raft fractions (fractions 2–3) of either CAV1 or CAV1 K176 mutant cells before treatment with doxorubicin compared with the cells that were treated with doxorubicin (right panel). After treatment with doxorubicin (0.5 μM) for 16 hours prior to fractionation, more P-glycoprotein was present in the extracts from CAV1 K176R mutant cells in the lipid raft fractions (fraction 2) than the extracts from cells that were transduced with wild-type CAV1 (right panel, Fig. 3B).

According to the literature, CAV1 can bind to P-glycoprotein and inhibit the transport activity of P-glycoprotein^24^,^25^. To evaluate the effect of the K176R mutant on the interaction between CAV1 and P-glycoprotein, we next examined their protein-protein interaction by immunoprecipitation after treatment with the anti-cancer drug doxorubicin. A physical interaction between overexpressed CAV1 and P-glycoprotein has been previously reported^24^, and we confirmed the physical interaction between CAV1 and P-glycoprotein in H460 cell lines that were transduced with V5-tagged CAV1 (Fig. 3A). Interestingly, no interaction was detected between the CAV1 K176R mutant and P-glycoprotein after treatment with doxorubicin. The CAV1 K176R mutant may abolish the inhibition of P-glycoprotein by CAV1 by reducing its interaction with P-glycoprotein.

P-glycoprotein is an active membrane transporter that is responsible for pumping numerous compounds out of cells, and its function is influenced by interacting proteins^26^. To gain further insight into the influence of the CAV1 K176R mutant on the intracellular distribution of P-glycoprotein, H460 cells transduced with either wild-type CAV1 or the K176R mutant were treated with doxorubicin and analyzed by subcellular fractionation with iodixanol velocity gradients (OptiPrep, Sigma-Aldrich) (Fig. 3B). Importantly, the distribution of P-glycoprotein in the CAV1 K176R mutant cells was significantly increased in the light buoyant density membrane fraction (fraction 2) after doxorubicin treatment. The subcellular distribution of wild-type CAV1 and the K176R mutant did not significantly differ, indicating that the K176R mutant did not influence the subcellular localization of CAV1.

**Figure 4:**
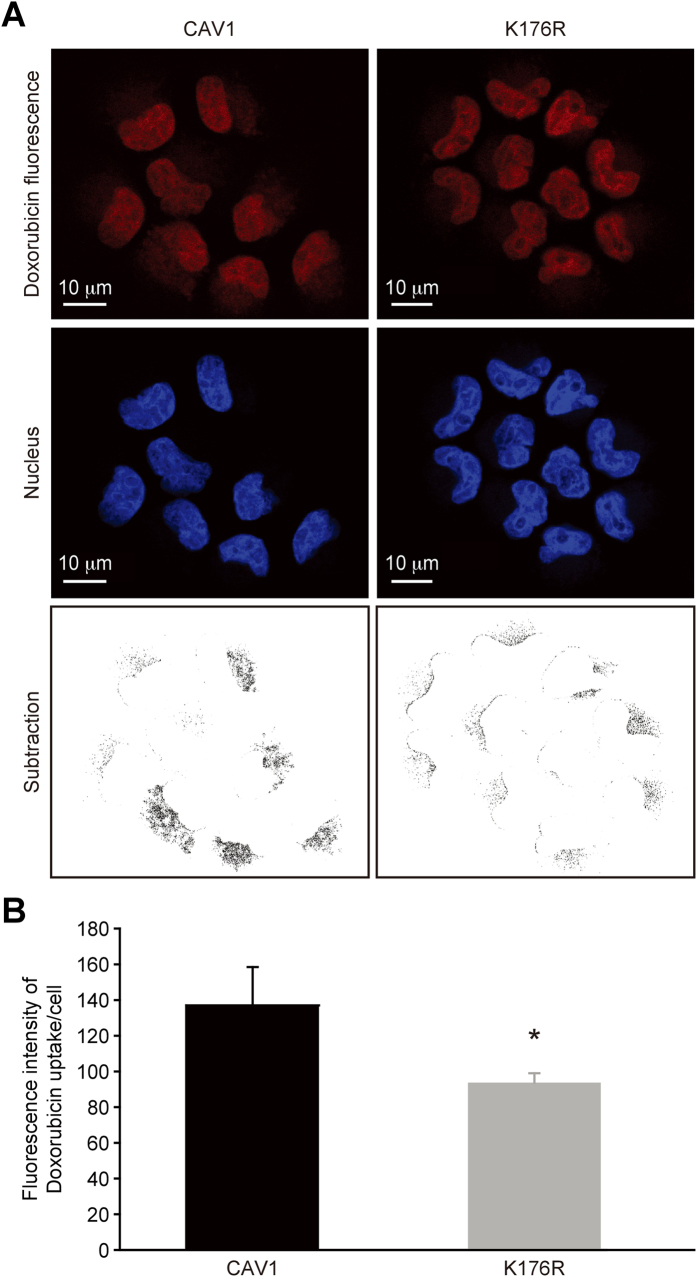
CAV1 K176R mutation decreased intracellular doxorubicin accumulation. (**A**) The fluorescence of doxorubicin in H460 cells that were transduced with CAV1 or the CAV1 K176R mutant and pretreated with 0.25 μM doxorubicin for 2 hours was analyzed by confocal microscopy. After the subtraction of the nuclear fluorescence, less doxorubicin fluorescence accumulated in the cytosol of cells that were transduced with the CAV1 K176R mutant. (**B**) Quantification of Intracellular doxorubicin accumulation. Error bars indicate the standard deviation. *p < 0.05.

The quantification of doxorubicin fluorescence by confocal microscopy can reportedly be used to assay the pumping function of P-glycoprotein^27^. To confirm the effect of the CAV1 K176R mutant on the pumping function of P-glycoprotein, we treated either wild-type CAV1- or K176R mutant-transduced H460 cells with doxorubicin and used confocal microscopy to measure the fluorescence of doxorubicin that was retained inside the cells^27^. We observed reduced intracellular fluorescence in cells that were transduced with the K176R mutant compared with cells that were transduced with wild-type CAV1 following treatment with doxorubicin. After the subtraction of fluorescent signals in the nucleus, the fluorescence of doxorubicin in cells that were transduced with the K176R mutant was lower than in cells that were transduced with wild-type CAV1 (Fig. 4A,B). In summary, the K176R mutant of CAV1 reduced the interaction between CAV1 and P-glycoprotein as well as the retention of doxorubicin in lung cancer cells, which could potentially transform cancer cells into MDR cells.

## Discussion

The failure of chemotherapy in cancer patients has been linked to the presence and development of drug resistance in cancer cells. Potential mechanisms of drug resistance in cancer cells involve the MDR phenotype, which includes the increased expression or enhanced function of multidrug transporters. The microenvironment of cell membranes, in which multidrug transporters are located, might also influence the function of multidrug transporters^28^,^29^. The essential protein CAV1 in caveolae has been shown to influence the MDR phenotype^30^. In the present study, we discovered that the K176R mutant of CAV1 decreased the formation of CAV1 oligomers. The C-terminal region (residues 151–178) of CAV1 has been reported to be an important domain for the formation of oligomer-oligomer interactions^4^. We also demonstrated that the CAV1 K176R mutant neutralized the inhibition of P-glycoprotein by CAV1. The development of MDR in cells is generally attributed to the increased surface expression of P-glycoprotein. Our data suggest that changes in the modification of CAV1 also play an important role in regulating the function of P-glycoprotein. This finding implies that the coordinated regulation of P-glycoprotein and CAV1 modification might be a general phenomenon in MDR cells.

In recent studies, CAV1 was found to colocalize and physically associate with P-glycoprotein between amino acid residues 36 and 44^11^,^18^. It was also reported that P-glycoprotein activity was negatively regulated by the interaction with CAV1^20^,^31^. Conversely, the mutation of the CAV1-binding motif of P-glycoprotein reduces the CAV1/P-glycoprotein interaction but also enhances the transport activity of P-glycoprotein^25^,^32^. In the present study, we found that the interaction between CAV1 and P-glycoprotein was enhanced by doxorubicin but weakened when lysine 176 of CAV1 was mutated to arginine. The drug-pumping function of P-glycoprotein was also enhanced in cells that were transduced with the CAV1 K176R mutant. We found that the lysine residue of CAV1 significantly impacted the CAV1-mediated regulation of drug transporters. Some studies have indicated that CAV1 overexpression enhances drug resistance, which could be attributed to the usage of different cell lines or drugs^18^. We cannot exclude the possibility that the CAV1 K176R mutant alters post-translational modifications, such as ubiquitination, sumoylation or phosphorylation, to indirectly promote the transport activity of P-glycoprotein. However, few studies have simultaneously analyzed the functionality of the CAV1 and P-glycoprotein interaction. The present findings indicate a novel mechanism for the regulation of P-glycoprotein via the post-translational modification of CAV1.

CAV1 is the major structural protein of caveolae. The levels of CAV1 correlate with the number of caveolae. Conflicting results have been reported regarding the role of caveolins in the development of MDR. Enhanced CAV1 expression has been observed in human cell lines with MDR phenotypes, including colchicine-resistant HT-29-MDR and Taxol-resistant lung carcinoma^33^,^34^. However, some MDR cells, such as J7.V1-1 and J7.T3-1.6, which are derived from murine macrophages, and Caco-V100, which is derived from human colon carcinoma cells, do not express high levels of CAV1 but express high levels of P-glycoprotein^34^,^35^. In our study, we found that CAV1 expression exerted a mild effect on the drug sensitivity of in cancer cells. However, the cell viability and drug accumulation in CAV1 K176R-transduced cells significantly differed from those of wild-type CAV1- or vector-transduced cells.

Barakat *et al.* reported that CAV1 overexpression would inhibit the activity of P-glycoprotein and phosphorylation of CAV1 of its tyrosine-14 by Src kinase, which would be involved in the modulation of p-glycoprotein transport activity^36^.We checked CAV1 phosphorylation with anti-phospho-CAV1 (Tyr14) antibody (#3251, Cell Signaling, Danvers, MA) in SDS-PAGE (data not shown). We found that CAV1 K176R mutant did not alter the status of CAV1 phosphorylation, which indicates that CAV1 K176R mutant did not influence the function of P-glycoprotein through altering the status of CAV1 phosphorylation.

In summary, our data show that the CAV1 K176R mutant blocks the interaction with P-glycoprotein and abolishes the inhibition of P-glycoprotein by CAV1. These effects contribute to our understanding of the regulation of P-glycoprotein and drug resistance in cancer cells.

## Methods

### Reagents and antibodies

Doxorubicin and *cis*-diamminedichloroplatinum (cisplatin; CDDP) were purchased from Selleck Chemicals (Houston, TX). Pacilitaxel was obtained from Calbiochem (Darmstadt, Germany). DMSO was purchased from Sigma. V5-conjugated agarose and the primary antibody against V5 were obtained from Bethyl Laboratories (Montgomery, TX). Anti-P-glycoprotein for flow cytometry was provided by Biolegend (San Diego, CA). Antibodies against GAPDH, Rab5 and Clathrin were obtained from Cell Signaling Technology (Danvers, MA); P-glycoprotein was provided with Merck Millipore (Darmstadt, Germany).

### Cell culture

Human non-small-cell lung carcinoma A549, H460 and RAW264.7 leukemia cells were cultured in RPMI-1640 medium (Invitrogen, Carlsbad, CA) supplemented with 10% fetal bovine serum (FBS, Hyclone, Waltham, MA), penicillin and streptomycin (Invitrogen, Carlsbad, CA) in an atmosphere of 5% CO_2_ at 37 °C. The 293T cells were cultured in Dulbecco’s Modified Eagle’s Medium (Invitrogen, Carlsbad, CA) supplemented with 10% FBS (Hyclone, Waltham, MA). Exponentially growing cells were used in all experiments.

### Plasmids

Full-length CAV1 cDNAs were amplified by PCR from mouse liver tissue and cloned into the pcDNA3.0 vector (Invitrogen, Carlsbad, CA) with the V5 tag to generate the pcDNA.V5-CAV1 construct. The pcDNA.V5-CAV1 lysine mutant constructs, lysine 176-to-arginine (K176R) and all lysine-to-arginine mutants (KR) were prepared using the QuickChange II Site-Directed Mutagenesis Kit (Stratagene, La Jolla, CA), and pcDNA.V5-CAV1 was used as the cDNA template. V5-CAV1 and V5-CAV1 K176R were subsequently cloned into the pLKO-AS2 lentivirus-based vector (National RNAi Core Facility, Academia Sinica, Taiwan) for lentiviral production.

### Lentivirus-mediated gene expression

HEK293T cells were transfected with either the AS2.V5-CAV1 or AS2.V5-CAV1 K176R constructs and the packaging plasmids pMD.G and pCMVR8.91 using Turbofect (Fermentas, Schwerte, Germany) according to the manufacturer’s directions. Culture medium containing the lentivirus was collected 48 and 72 hours after transfection. H460, A549 and RAW264.7 cells were infected overnight with lentiviruses in the presence of 8 μg/ml polybrene (Sigma-Aldrich, St. Louis, MO) and cultured in fresh medium for another 24 h. The infected cells were selected in medium containing 400–1000 μg/ml puromycin until the uninfected cells were completely killed. The stable colonies were pooled together for further experiments.

### Immunoprecipitation and immunoblotting

For oligomer detection, whole cells were lysed in 2% SDS buffer with 3% 2-mercaptoethanol and loaded in SDS PAGE without prior heating. Cell lysates were harvested and lysed in ice-cold lysis buffer containing 150 mM NaCl, 20 mM Tris-HCl, 1% Triton X-100, and protease and phosphatase inhibitor cocktail (Roche, Basel, Switzerland) and homogenized on ice in 1.5-ml microfuge tubes by sonication for 30 seconds. The protein concentrations were determined using the Bradford assay (Bio-Rad, Hercules, CA). Cellular extracts (200μg) were incubated with the indicated antibodies overnight, followed by incubation with Protein A/G beads for 1 hour or antibody-conjugated beads overnight at 4 °C. The immunocomplexes were separated by SDS-PAGE and transferred to nitrocellulose membranes. The membranes were then incubated with the indicated primary antibodies followed by incubation with an HRP-conjugated secondary antibody. Immunoreactive bands were detected using Western Lighting® Plus-ECL (PerkinElmer, Waltham, MA).

### Drug efflux assay of transduced cells

The cells were trypsinized, resuspended at 1 × 10^6^cells/ml in phenol red-free medium and incubated with the fluorescent probe eFluxx-ID Gold (ENZO Life Sciences, Lörrach, Germany) for 30 min at 37 °C according to manufacturer’s recommendations in the presence of 5 mM verapamil, 10 mM MK-571, and 50 mM novobiocin. The samples were then analyzed immediately by flow cytometry. The eFluxx-ID Gold (λ_ex/em_ = 530/555 nm) fluorescence intensity was measured using the FL2/PE (585/42 nm filter) channel. The gating of viable cells was based on the light scatter parameters.

### Cell viability assay

H460 and A549 cells were plated in triplicate in 96-well plates at a density of 5 × 10^3^ cells/well. After the cells had adhered to the plates, the drugs were individually added to the experimental groups at the final indicated concentration, and the same amount of drug dissolution medium was added to the control group. After 24 hours, the cell viability was evaluated using the WST1 (Roche Applied Science) viability assay. The viability was calculated as the ratio of the OD_450_ of treated cells to the OD_450_ of untreated control cells.

### Immunofluorescent flow cytometry of surface P-glycoprotein

Cells (5 × 10^5^ per sample) were collected by centrifugation, washed with cold PBS and incubated with 100 μl of the corresponding primary antibody (5 μg/mL) for 1 hour. Post-incubation, the cells were washed twice with PBS/0.05% BSA, stained with secondary antibody at a 1:1000dilution for 30 min at 4 °C, washed again, re-suspended in PBS/2% paraformaldehyde and analyzed by flow cytometry. Cells stained with isotype-matched antibody or with secondary antibody alone were used as controls.

### Preparation of subcellular fractions

Cells were scraped into ice-cold 0.5 M sodium bicarbonate and lysed by 20 strokes in a Dounce homogenizer followed by 10 passes through a 21-gauge needle. The membrane fraction supernatant was separated in a 0–40% Opti-Prep gradient layer (60% iodixanol, Sigma, St. Louis, MO) and then centrifuged for 4 hours at 20,0000 g in an SW41 Ti swinging bucket rotor centrifuge. A band of membranes was visible just below the 5% Opti-prep layer. This material was collected and used as the lipid raft fraction.

### Doxorubicin Intracellular Distribution and Accumulation

CAV1- and CAV1 K176R-expression H460 cells were seeded on coverslips in six-well plates and allowed to grow overnight. On the following day, the cells were washed with PBS, treated with 2.5 μM doxorubicin for 2 hours, and then examined by confocal microscopy. Doxorubicin accumulation was determined using ImageJ.

### Statistics

All data are expressed as the mean ± standard deviation of three or more experiments and were statistically evaluated using Student’s *t* test. Differences were considered significant at *p* < 0.05.

## Additional Information

**How to cite this article**: Lee, C.-Y. *et al.* The influence of a caveolin-1 mutant on the function of P-glycoprotein. *Sci. Rep.* **6**, 20486; doi: 10.1038/srep20486 (2016).

## References

[b1] Brown D. A. Lipid rafts, detergent-resistant membranes, and raft targeting signals. Physiology (Bethesda) 21, 43–9 (2006). 10.1152/physiol.00032.2006 17119156

[b2] Smart E. J. *et al.* Caveolins, liquid-ordered domains, and signal transduction. Mol. Cell. Biol. 19, 7289–304 (1999). 10523618 10.1128/mcb.19.11.7289 PMC84723

[b3] Couet J., Shengwen L., Okamoto T., Scherer P. E. & Lisanti M. P. Molecular and cellular biology of caveolae paradoxes and plasticities. Trends Cardiovasc. Med. 7, 103–10 (1997). 21235872 10.1016/S1050-1738(97)00001-7

[b4] Schlegel A. & Lisanti M. P. A molecular dissection of caveolin-1 membrane attachment and oligomerization. Two separate regions of the caveolin-1 C-terminal domain mediate membrane binding and oligomer/oligomer interactions *in vivo*. J. Biol. Chem. 275, 21605–17 (2000). 10801850 10.1074/jbc.M002558200

[b5] Ritz D. *et al.* Endolysosomal sorting of ubiquitylated caveolin-1 is regulated by VCP and UBXD1 and impaired by VCP disease mutations. Nat. Cell. Biol. 13, 1116–23 (2011). 21822278 10.1038/ncb2301 PMC3246400

[b6] Kim W. *et al.* Systematic and quantitative assessment of the ubiquitin-modified proteome. Mol. Cell. 44, 325–40 (2011). 21906983 10.1016/j.molcel.2011.08.025 PMC3200427

[b7] Lavie Y., Fiucci G. & Liscovitch M. Up-regulation of caveolae and caveolar constituents in multidrug-resistant cancer cells. J Biol Chem 273, 32380–3 (1998). 9829965 10.1074/jbc.273.49.32380

[b8] Davidson B. *et al.* Caveolin-1 expression in ovarian carcinoma is MDR1 independent. Am. J. Clin. Pathol. 117, 225–34 (2002). 11863219 10.1309/u40r-1bn4-6kj3-bdg3

[b9] Cai C. & Chen J. Overexpression of caveolin-1 induces alteration of multidrug resistance in Hs578T breast adenocarcinoma cells. Int. J. Cancer. 111, 522–9 (2004). 15239129 10.1002/ijc.20300

[b10] Higgins C. F. ABC transporters: from microorganisms to man. Annu. Rev. Cell. Biol. 8, 67–113 (1992). 1282354 10.1146/annurev.cb.08.110192.000435

[b11] Demeule M., Jodoin J., Gingras D. & Beliveau R. P-glycoprotein is localized in caveolae in resistant cells and in brain capillaries. FEBS Lett. 466, 219–24 (2000). 10682831 10.1016/s0014-5793(00)01087-5

[b12] Hehlgans S. & Cordes N. Caveolin-1: an essential modulator of cancer cell radio-and chemoresistance. Am. J. Cancer Res. 1, 521–30 (2011). 21984970 PMC3186050

[b13] Kirchner P., Bug M. & Meyer H. Ubiquitination of the N-terminal region of caveolin-1 regulates endosomal sorting by the VCP/p97 AAA-ATPase. J. Biol. Chem. 288, 7363–72 (2013). 23335559 10.1074/jbc.M112.429076 PMC3591644

[b14] Hayer A. *et al.* Caveolin-1 is ubiquitinated and targeted to intralumenal vesicles in endolysosomes for degradation. J. Cell. Biol. 191, 615–29 (2010). 21041450 10.1083/jcb.201003086 PMC3003328

[b15] Sargiacomo M. *et al.* Oligomeric structure of caveolin: implications for caveolae membrane organization. Proc. Natl. Acad. Sci. USA 92, 9407–11 (1995). 7568142 10.1073/pnas.92.20.9407 PMC40994

[b16] Lei M. G., Tan X., Qureshi N. & Morrison D. C. Regulation of cellular caveolin-1 protein expression in murine macrophages by microbial products. Infect. Immun. 73, 8136–43 (2005). 16299308 10.1128/IAI.73.12.8136-8143.2005 PMC1307083

[b17] Fletcher J. I., Haber M., Henderson M. J. & Norris M. D. ABC transporters in cancer: more than just drug efflux pumps. Nat. Rev. Cancer. 10, 147–156 (2010). 20075923 10.1038/nrc2789

[b18] Engelman J. A. *et al.* Recombinant expression of caveolin-1 in oncogenically transformed cells abrogates anchorage-independent growth. J. Biol. Chem. 272, 16374–81 (1997). 9195944 10.1074/jbc.272.26.16374

[b19] Cai C., Zhu H. & Chen J. Overexpression of caveolin-1 increases plasma membrane fluidity and reduces P-glycoprotein function in Hs578T/Dox. Biochem. Biophys. Res. Commun. 320, 868–74 (2004). 15240128 10.1016/j.bbrc.2004.06.030

[b20] Bosch I. & Croop J. P-glycoprotein multidrug resistance and cancer. Biochim. Biophys. Acta. 1288, F37–54 (1996). 8876632 10.1016/0304-419x(96)00022-4

[b21] Stordal B. *et al.* Resistance to paclitaxel in a cisplatin-resistant ovarian cancer cell line is mediated by P-glycoprotein. PLoS One. 7, e40717 (2012). 22792399 10.1371/journal.pone.0040717 PMC3394717

[b22] Wang J. *et al.* Down-regulation of P-glycoprotein is associated with resistance to cisplatin and VP-16 in human lung cancer cell lines. Anticancer Res. 30, 3593–8 (2010). 20944142

[b23] Barakat S. *et al.* Modulation of p-glycoprotein function by caveolin-1 phosphorylation. J. Neurochem. 101, 1–8 (2007). 17326770 10.1111/j.1471-4159.2006.04410.x

[b24] Barakat S. *et al.* Regulation of brain endothelial cells migration and angiogenesis by P-glycoprotein/caveolin-1 interaction. Biochem. Biophys. Res. Commun. 372, 440–6 (2008). 18485890 10.1016/j.bbrc.2008.05.012

[b25] Orlowski S., Martin S. & Escargueil A. P-glycoprotein and ‘lipid rafts’: some ambiguous mutual relationships (floating on them, building them or meeting them by chance?). Cell. Mol. Life. Sci. 63, 1038–59 (2006). 16721513 10.1007/s00018-005-5554-9 PMC11136201

[b26] Shen F. *et al.* Quantitation of doxorubicin uptake, efflux, and modulation of multidrug resistance (MDR) in MDR human cancer cells. J. Pharmacol. Exp. Ther. 324, 95–102 (2008). 17947497 10.1124/jpet.107.127704

[b27] Veldman R. J. *et al.* Altered sphingolipid metabolism in multidrug-resistant ovarian cancer cells is due to uncoupling of glycolipid biosynthesis in the Golgi apparatus. FASEB J. 16, 1111–3 (2002). 12039850 10.1096/fj.01-0863fje

[b28] Sietsma H., Veldman R. J. & Kok J. W. The involvement of sphingolipids in multidrug resistance. J. Membr. Biol. 181, 153–62 (2001). 11420602 10.1007/s00232-001-0033-1

[b29] Lavie Y., Fiucci G. & Liscovitch M. Upregulation of caveolin in multidrug resistant cancer cells: functional implications. Adv. Drug Deliv. Rev. 49, 317–23 (2001). 11551402 10.1016/s0169-409x(01)00144-2

[b30] Zhu H., Cai C. & Chen J. Suppression of P-glycoprotein gene expression in Hs578T/Dox by the overexpression of caveolin-1. FEBS Lett 576, 369–74 (2004). 15498565 10.1016/j.febslet.2004.09.041

[b31] Jodoin J. *et al.* P-glycoprotein in blood-brain barrier endothelial cells: interaction and oligomerization with caveolins. J. Neurochem. 87, 1010–23 (2003). 14622130 10.1046/j.1471-4159.2003.02081.x

[b32] Okamoto T., Schlegel A., Scherer P. E. & Lisanti M. P. Caveolins, a family of scaffolding proteins for organizing “preassembled signaling complexes” at the plasma membrane. J. Biol. Chem. 273, 5419–22 (1998). 9488658 10.1074/jbc.273.10.5419

[b33] Yang C. P., Galbiati F., Volonte D., Horwitz S. B. & Lisanti M. P. Upregulation of caveolin-1 and caveolae organelles in Taxol-resistant A549 cells. FEBS Lett. 439, 368–72 (1998). 9845355 10.1016/s0014-5793(98)01354-4

[b34] Greenberger L. M., Lothstein L., Williams S. S. & Horwitz S. B. Distinct P-glycoprotein precursors are overproduced in independently isolated drug-resistant cell lines. Proc. Natl. Acad. Sci. USA 85, 3762–6 (1988). 2897689 10.1073/pnas.85.11.3762 PMC280298

[b35] Haber M. *et al.* Altered expression of M beta 2, the class II beta-tubulin isotype, in a murine J774.2 cell line with a high level of taxol resistance. J. Biol. Chem. 270, 31269–75 (1995). 8537394 10.1074/jbc.270.52.31269

[b36] Barakat S. *et al.* Modulation of p-glycoprotein function by caveolin-1 phosphorylation. J Neurochem 101, 1–8 (2007). 17326770 10.1111/j.1471-4159.2006.04410.x

